# The Book World of Medicine and Science

**Published:** 1897-06-26

**Authors:** 


### The Book World of Medicine and Science.

Diseases of the Ear, Nose, and Throat. By Seth Scott Bishop, M.D., LL.D. Illustrated with 100 coloured lithographs and 168 additional illustrations. (Philadelphia: The F. A. Davis Co. 1897. Price 4 dols.)

This is what is called a condensed text-book, and the term is fairly expressive of the character of the book. The great extent of the subjects dealt with and the large amount of literature to which they have given rise, make it impossible to give in one volume a complete account of all that has been done in the various departments into which these specialties have already become subdivided. But the very fact that the literature is so extensive makes it all the more necessary, both to the student and the practitioner, to be provided with such a book as this, which, although the author modestly speaks of it as a key or introduction to the exhaustive treatises already in the field, is really itself a very useful treatise, giving a good and well-illustrated description of the diseases of the regions named. The reader will observe a certain unevenness in the allocation of the pages to the various subjects. In regard to this, however, the author explains that in such matters as the serum treatment of diphtheria, the management of mastoid disease, the treatment of hay fever, and the improvements which have been recently introduced into compressed air instruments, vaporisers, &c., there is but little quite recent information to be found in books of similar nature to this, and on referring to the pages in question we cannot but think that the author has done well to give to these matters a somewhat greater prominence than quite accords with absolute symmetry of arrangement. Nevertheless, we do not at present feel inclined to endorse all that is said in regard to the use of compressed air in dealing with middle ear disease. Undoubtedly there are great advantages in being able to ensure a steady and well-maintained pressure. On the other hand, it is difficult to believe that some of the pressures mentioned can be safe. So long as a catheter does not quite fit there is always some escape of air, and thus the pressure within the tympanum never rises at all near to that in the bag; yet even with the comparatively limited amount of air contained in the hand bags in common use it is well known that accidents have happened. With a large reservoir, however, of highly compressed air in reserve it would be possible, if by chance the catheter fitted, to bring nearly the whole of the pressure to bear within the tympanum, and one cannot avoid the conclusion that these powerful instruments in any but the most careful hands might be productive of mischief. On the other hand it must be borne in mind that the compressed air apparatus does undoubtedly render it possible to obtain access to the tympanum in the great majority of cases without any catheterisation, and with much less distress to the patient than by the ordinary methods of politzerisation; and for the production of sprays and such like forms of local medication the reservoir of compressed air certainly offers many and great advantages.

Comparing the different portions into which the book is divided, that which is devoted to diseases of the ear appears to be most complete, and that which deals with diseases of the larynx the least so. With the exception of the laryngeal diseases, of which it gives but a sketchy presentment, the book may be thoroughly recommended as giving a practical review of the subjects with which it deals. The illustrations are very good, and are made use of in such a manner as to elucidate the meaning of the author; at the same time it must be confessed that the coloured lithographs are really diagrammatic rather than representative. A lecturer with coloured chalks can often more easily make his meaning clear than one with white chalk only, and in this sense the colouring of the illustrations is very useful, but we should not like to admit that they are representative of anything that occurs in nature. There can be no doubt that this book will be of great use to those for whom it has been written—students and "progressive practitioners" rather than specialists.

Burdett's Hospitals and Charities, 1897. By Henry C. Burdett, K.C.B. (London: The Scientific Press, pp. 918. Price 5s.)

This is something more than a year-book of hospitals; it is, as is stated on the cover, a Year-Book of Philanthropy. No doubt the hospitals bulk largely in the objects dealt with, but many other subjects are included, such as lunatic asylums, convalescent homes, nursing institutions, missionary and religious societies, orphanages, homes, and institutions for the blind, the deaf and dumb, and the incurable. All these are passed in review, and to those interested in the various branches of philanthropic effort such a work must be an almost necessary handbook and companion. A considerable proportion of the pages are occupied by condensed and carefully classified information regarding the individual institutions dealt with, the number of which is legion. The general plan is to give as to each hospital the date of its establishment, the nature of its governing body, the qualifications of its governors, the names of the president, chairman of committee, treasurer, secretary, and medical and nursing staffs, the number of beds and the average number occupied, the number of in and out-patients, the terms of admission, the income and expenditure, and, where possible, the visiting days. The same system is maintained in regard to a large number of the hospitals in the colonies and abroad, although in some cases the amount of information given is not quite so extensive. On the other hand, the larger institutions are dealt with much more fully, the days and hours of attendance of the various members of the medical staff, for example, being given in regard to many of the London hospitals.

While one of the main objects of the book is to serve as an authentic and reliable directory, many of its pages are devoted to general information concerning charities, to criticism of their methods, and to a careful tabulation and comparison of their results and of the details of their working.

A comparison with former volumes shows that while the general plan of the directory portion is maintained, the critical section is practically a new book each year. A very interesting feature in this edition is the careful analysis which is made of the various items of hospital expenditure which may be classed under the term commissariat, showing, as it does, how much a hospital may lose by laxity in regard to small and apparently trivial matters affecting the food supply. In such matters every minute saving and every loss, however fractional, has to be multiplied both by the number of the patients and by the days in the year, so that farthings become matters of immense importance.

Turning to the other side of hospital finance, viz., the collecting of the funds, we find on the one hand strong denunciations of street collections, as to the great evils of which most people are now agreed, and on the other many expressions of buoyant hopefulness as to the almost unlimited depth of John Bull's pocket if he is but asked in the right way and the request is made to the right people. While candidly critical as to defects in method, this year-book of philanthropy gives good grounds for confidence in the stability of our system of supporting charities by voluntary contributions.

